# Reduced glutathione ameliorates sepsis-induced acute kidney injury in mouse model: involvement of cuproptosis

**DOI:** 10.1080/0886022X.2025.2518227

**Published:** 2025-06-23

**Authors:** Lu Wang, Long Jiang, Jingjing Wang, Wen Tang, Zhigao Wang, Rennan Guo, Daquan Zhang

**Affiliations:** Department of Critical Care Medicine, People’s Hospital of Xinjiang Uygur Autonomous Region, Urumqi, Xinjiang Uygur Autonomous Region, China

**Keywords:** Reduced GSH, AKI, sepsis cuproptosis, CLP

## Abstract

**Background:**

Cuproptosis, a copper-induced form of programmed cell death, has been implicated in the pathogenesis of acute kidney injury (AKI) and sepsis. Reduced glutathione (GSH), a potent antioxidant, exhibits both anti-inflammatory and anti-oxidative properties. However, its potential role in modulating cuproptosis in AKI remains unexplored. This study investigates the effects of reduced GSH on cuproptosis in a sepsis-induced AKI mouse model.

**Methods:**

A sepsis mouse model was established *via* cecal ligation and puncture (CLP). Reduced GSH was administered to CLP-induced mice. Survival rates, bacterial load, inflammatory cytokine levels, oxidative stress markers, kidney function, copper (Cu) levels, and the expression of SLC31A1 were evaluated.

**Results:**

Reduced GSH treatment significantly improved survival rates and reduced bacterial loads in CLP mice. Additionally, reduced GSH attenuated inflammation and oxidative stress in the septic mice. It restored kidney function, decreased Cu levels in both urine and kidney tissues, and downregulated the expression of SLC31A1.

**Conclusion:**

Reduced GSH ameliorates sepsis-induced AKI by suppressing cuproptosis, offering potential therapeutic implications for the management of AKI in septic conditions.

## Introduction

Acute kidney injury (AKI), also known as acute renal failure, is associated with significant morbidity and mortality [1]. AKI is a serious condition triggered by various factors, including dehydration, severe infections, certain medications, or trauma [2]. Sepsis, a life-threatening condition resulting from the extreme response of body to infection, can lead to widespread inflammation. This inflammation triggers a cascade of events that damage various organs, including the kidneys, and often culminates in SKI [3]. The hallmark characteristics of AKI include reduced urine output, elevated blood urea nitrogen (BUN) and creatinine levels, and fluid retention [4]. Treatment strategies for AKI primarily focus on addressing the underlying cause, managing complications, and providing supportive care to promote kidney recovery [5]. However, the effectiveness of current treatments remains limited. Therefore, it is crucial to deepen our understanding of the pathogenesis of AKI and explore novel therapeutic approaches.

Cuproptosis is a distinct form of cell death triggered by the accumulation of copper (Cu) in mitochondria. Excess Cu accumulation promotes the aggregation of lipoylated dihydrolipoamide S-acetyltransferase (DLAT) and other lipoylated tricarboxylic acid (TCA) cycle proteins, leading to the loss of iron-sulfur cluster proteins, proteotoxic stress, and ultimately cell death [6]. Cuproptosis has been implicated in various diseases, including cancers [7], osteoarthritis [8], and sepsis [9]. Notably, Qiu et al. demonstrated that suppressing the expression of SLC31A1, a key mediator of cellular Cu uptake in eukaryotic cells [10], rescued renal function, improved mitochondrial activity, and inhibited apoptosis in AKI mice [11]. These findings suggest that there is a correlation between SLC31A1 suppression and reduced cuproptosis and highlight that cuproptosis and SLC31A1 could be promising targets for the treatment of AKI.

Reduced glutathione (GSH) is a vital antioxidant that functions both directly as a free radical scavenger and as a cofactor for antioxidant enzymes, protecting cells from oxidative damage and pro-oxidants [12]. Reduced GSH has been used to treat various conditions, including liver diseases [13], brain diseases [14], and kidney diseases [15]. Previous studies showed that reduced GSH ameliorated AKI by inhibiting ferroptosis [16]. However, the specific effects of reduced GSH on cuproptosis in AKI remain unclear. In this study, we investigated the role of reduced GSH in modulating cuproptosis in AKI using a sepsis-induced mouse model.

## Materials and methods

### Mice model of sepsis

This animal study was approved by the People’s Hospital of Xinjiang Uygur Autonomous Region. This study was performed in strict accordance with the Guide for the Care and Use of Laboratory Animals (8th edition, NIH). Eight-week-old male C57BL/6 mice were obtained from Vital River Laboratory Animal Technology Co., Ltd. (Beijing). Sepsis-associated acute kidney injury (AKI) was induced using the cecal ligation and puncture (CLP) method [17]. Mice were anesthetized *via* intraperitoneal injection of pentobarbital (50 mg/kg). Mice were sacrificed *via* intraperitoneal injection of pentobarbital sodium solution at a dose of 120 mg/kg. A 1 cm midline incision was made in the ventral abdomen to expose the cecum. The distal three-fourths of the cecum was ligated using 4–0 silk sutures. To induce sepsis, a double puncture was performed on the ligated cecum using a 20 G needle, and a small amount of cecal contents was gently extruded. The peritoneum and skin were then closed with 4–0 silk sutures. Following surgery, the mice received subcutaneous injection of 1 mL pre-warmed sterile saline for resuscitation. In the sham group, the same surgical procedure was performed, excluding the ligation and puncture of the cecum.

### Experimental design

Three groups of mice (20 mice per group) were established for this study: the Sham group, the Vehicle group (septic mice treated with PBS), and the RGSH group (septic mice treated with reduced glutathione). L-glutathione reduced was purchased from Sigma and dissolved in PBS.

Septic mice were treated with either PBS or reduced glutathione (800 mg/kg) *via* intraperitoneal injection daily for one week prior to the CLP procedure according to a previous study [16]. Additional treatments were administered at 4, 12, 24, and 48 h post-CLP. Mice survival was monitored every two hours following CLP. Samples were collected 24 h after sepsis induction for further analysis.

### Bacterial burden

Twenty-four hours post-CLP, kidney tissues were harvested and homogenized in 2 mL of PBS. The homogenates were centrifuged for 5 min, and the supernatants were diluted appropriately. The diluted homogenates were then evenly spread on nutrient agar plates. After incubation at 37 °C for 24 h, bacterial colonies were counted.

### Kidney function

Serum creatinine (Scr) and blood urea nitrogen (BUN) levels were measured using the Creatinine Assay Kit and Urea Assay Kit, respectively (both from Abcam). Urinary kidney injury molecule 1 (KIM-1) levels were quantified using the KIM-1 ELISA Kit (Abcam).

### Elisa

Serum levels of pro-inflammatory cytokines, including IL-1β, IL-6, and TNF-α, were measured using commercial ELISA kits (Abcam) according to the manufacturer’s instructions.

### H&E staining

Mouse kidneys were harvested and fixed in paraformaldehyde. The paraffin-embedded tissues were sectioned into 3 µm slices. Hematoxylin and eosin (H&E) staining was performed to evaluate tissue morphology. Tubular injury was assessed semiquantitatively using a grading scale from 0 to 5, where Grade 0 indicates normal kidney tissue, and Grades 1 through 5 correspond to the percentage of tubular damage: 1 (≤10%), 2 (11–25%), 3 (26–45%), 4 (46–75%), and 5 (≥76%) [18]. The assessment of tubular injury was conducted by experienced histopathologists who were blinded to the experimental group assignments. This blinding process was implemented to eliminate potential bias in the scoring of tissue samples, ensuring objective evaluation of tubular damage across all experimental conditions

### Biochemical analysis

The levels of malondialdehyde (MDA) and the activities of adenosine triphosphatase (ATP) and manganese superoxide dismutase (MnSOD) in kidney tissues were measured using the MDA Assay Kit, ATP Assay Kit, and SOD Assay Kit, respectively (all from Abcam).

### Immunohistochemical staining

After dewaxing, rehydration, and antigen retrieval, tissue sections were treated with 3% H_2_O_2_ to block endogenous peroxidase activity and then blocked with 5% goat serum. The sections were incubated overnight at 4 °C with an anti-SLC31A1 antibody (1:2000, Thermo Fisher). The following day, the appropriate secondary antibody was applied, and the reaction was visualized using DAB (3,3′-diaminobenzidine). The stained sections were analyzed under a light microscope.

### Western blot

Western blotting was performed following a standard protocol as described previously [19]. Briefly, equal amounts of tissue homogenates were loaded onto SDS-PAGE gels and subsequently transferred to PVDF membranes. The membranes were blocked with 5% nonfat milk and incubated overnight with primary antibodies. The primary antibodies used in this study were as follows: anti-SLC31A1 (1:5000, Thermo Fisher), anti-HSP70 (1:1000, Abcam), anti-FDX1 (1:2000, Abcam), and anti-ACO2 (1:1000, Abcam). Immunoreactive bands were visualized using an enhanced chemiluminescence (ECL) substrate. Band intensities were quantified using ImageJ software.

### Real-time PCR

Total RNAs were extracted using TRIZOL reagent (Thermo Fisher). cDNA was synthesized using First Strand cDNA Synthesis Kit. Quantitative PCR was performed using Power SYBR^™^ Green PCR Master Mix and QuantStudio Real-Time PCR system. Primers used in this study were: Mouse SLC31A1 Forward (5′-3′) GGGGCTTACCCTGTGAAGACTTT, Reverse (5′-3′) CGTCCGTGTGGTTCATACCC. Mouse GAPDH Forward (5′-3′) CGAGAATGGGAAGCTTGTCATC, Reverse (5′-3′) CGGCCTCACCCCATTTG. GAPDH was used as internal control and the expression was analyzed using the 2^- ΔΔCt^ method.

### Icp-ms

Copper (Cu) levels in the samples were measured using inductively coupled plasma mass spectrometry (ICP-MS, Agilent 7700X) following the previously described protocol [19].

### Statistical analysis

Prior to statistical analysis, we assessed the normality of our data using the Shapiro-Wilk test (*n* ≥ 3) for all datasets. After confirming that our data met the assumptions of normality, we proceeded with parametric tests. For comparisons between multiple groups, we employed Brown-Forsythe ANOVA followed by Dunnett’s T3 multiple comparisons test. This approach was chosen to account for potential heteroscedasticity in our data while maintaining robust statistical power. All statistical analyses were performed using GraphPad Prism 5 (GraphPad Software, San Diego, CA, USA). Results are presented as means ± standard deviation (SD). A p-value < 0.05 was considered statistically significant.

## Results

### Reduced glutathione prevented sepsis-induced death and decreased bacterial burden in blood and kidney after CLP surgery

**Figure 1.**
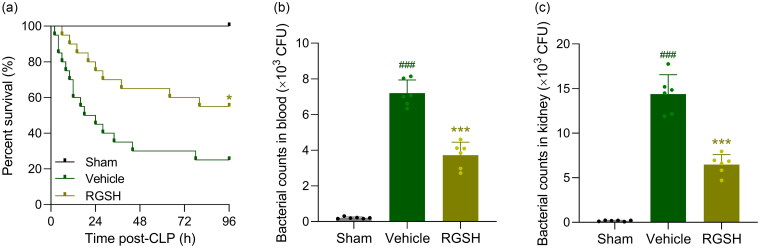
Reduced glutathione ameliorated sepsis-induced death and bacterial growth in blood and kidney after CLP surgery. a, overall survival of mice during 96 h recording post-CLP from each group. *n* = 20 for each group. *p < 0.05 compared to vehicle. Log-rank (Mantel-Cox) test. b and c, bacterial counts in blood and kidney at 24 h after CLP surgery. *n* = 6 for each group. ^###^*p* < 0.001 compared to sham and ***p < 0.001 compared to vehicle.

We first investigated the effects of reduced glutathione on sepsis-induced mortality in mice. As shown in Figure 1a, CLP surgery resulted in severe mortality, with only approximately 20% of mice surviving at 96 h after the procedure. In contrast, mice treated with reduced glutathione exhibited a significantly higher survival rate compared to the vehicle-treated group, suggesting that reduced glutathione offered protection against CLP-induced death.

Next, we examined bacterial burden. CLP mice had exhibited significantly higher bacterial loads in both blood (Figure 1b) and kidney (Figure 1c) compared to sham mice. Interestingly, treatment with reduced glutathione markedly reduced bacterial burdens in both blood and kidney tissues.

Taken together, these findings indicate that reduced glutathione protects mice from CLP-induced sepsis by reducing bacterial burden and improving survival. Importantly, our findings demonstrated that reduced glutathione treatment in healthy mice did not affect body weight, suggesting its potential safety as a therapeutic intervention (Figure S1a). Furthermore, the absence of changes in serum LDH, BUN, and creatinine levels in treated mice provides additional evidence supporting the safety profile of reduced glutathione (Figure S1b–d). These results collectively indicate that reduced glutathione may be a promising and well-tolerated treatment option, though further studies are necessary to fully establish its safety and efficacy in various clinical applications.

### Reduced glutathione suppressed inflammation in CLP mice

**Figure 2.**
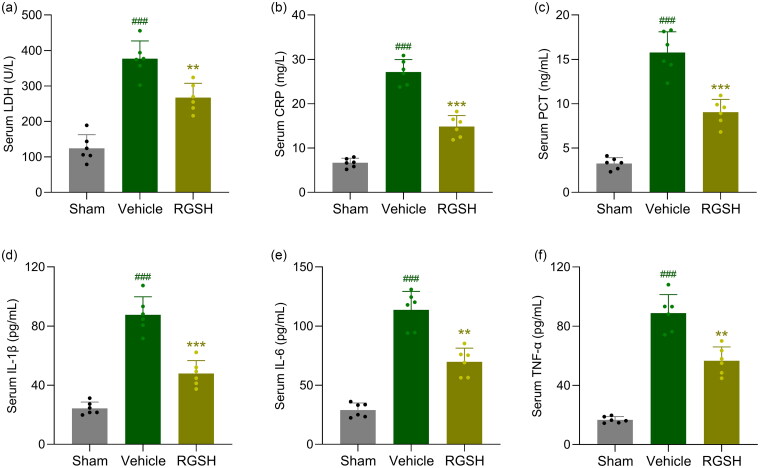
Reduced glutathione ameliorated infection indexes and inflammatory cytokines in serum after CLP surgery. Levels of LDH (a), CRP (b), PCT (c), IL-1β (d), IL-6 (e) and TNF-α (f) in serum at 24 h after CLP surgery were compared. *n* = 6 for each group. ^###^*p* < 0.001 compared to sham and **p < 0.01, ***p < 0.001 compared to vehicle.

To evaluate the effects of reduced glutathione on inflammation in CLP mice, we analyzed serum levels of infection markers (LDH, CRP, and PCT) and inflammatory cytokines (IL-1β, IL-6, and TNF-α). CLP mice showed significantly elevated levels of LDH (Figure 2a), CRP (Figure 2b), PCT (Figure 2c), IL-1β (Figure 2d), IL-6 (Figure 2e), and TNF-α (Figure 2f) compared to sham mice.

Notably, treatment with reduced glutathione significantly reduced the levels of these markers and cytokines in CLP mice. These results suggest that reduced glutathione effectively attenuates infection and inflammation in CLP mice.

### Reduced glutathione ameliorated acute kidney injury in CLP mice

**Figure 3.**
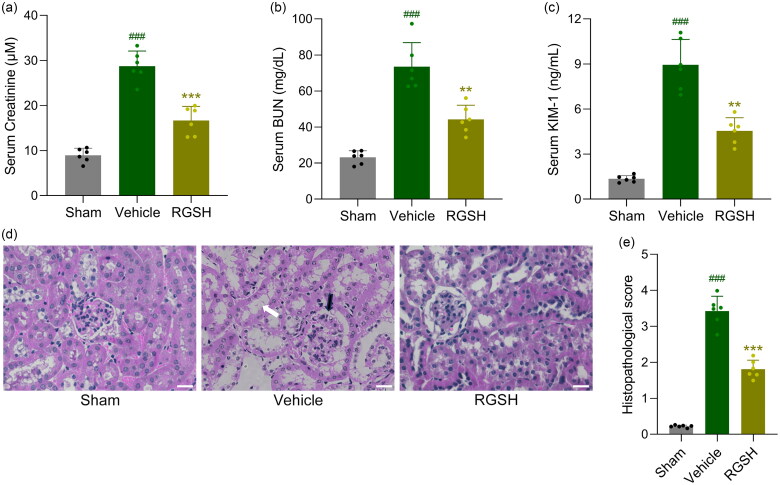
Reduced glutathione ameliorated sepsis-induced acute kidney injury in mice. Serum levels of creatinine (a), BUN (b) and KIM-1 (c) at 24 h after CLP surgery were compared. d, representative HE staining among different groups and the comparisons of histopathological score (e, black arrow: glomerulus degeneration, white arrow: renal tubules edema). Scale bar = 20 μm. *n* = 6 for each group. ^###^*p* < 0.001 compared to sham and **p < 0.01, ***p < 0.001 compared to vehicle.

The effects of reduced glutathione on kidney injury were evaluated by measuring serum levels of kidney function markers, including creatinine, BUN, and KIM-1. As shown in Figure 3a, creatinine levels were significantly elevated in CLP mice compared to sham controls. Interestingly, CLP mice treated with reduced glutathione showed significantly lower serum creatinine levels compared to vehicle-treated CLP mice. Similarly, reduced glutathione treatment significantly decreased serum BUN (Figure 3b) and KIM-1 levels (Figure 3c) in CLP mice. These findings indicate that reduced glutathione improves kidney function in CLP mice.

Histopathological analysis further corroborated these results. CLP mice exhibited pronounced histological changes in kidney tissues, with significantly higher histopathological scores compared to sham mice (Figure 3d and e). In contrast, reduced glutathione-treated CLP mice displayed markedly reduced histological abnormalities and significantly lower histopathological scores compared to vehicle-treated CLP mice (Figure 3d and e).

Collectively, these findings demonstrate that reduced glutathione effectively rescues kidney function and mitigates kidney damage in CLP mice.

### Reduced glutathione inhibited inflammation and oxidative stress in CLP mice

**Figure 4.**
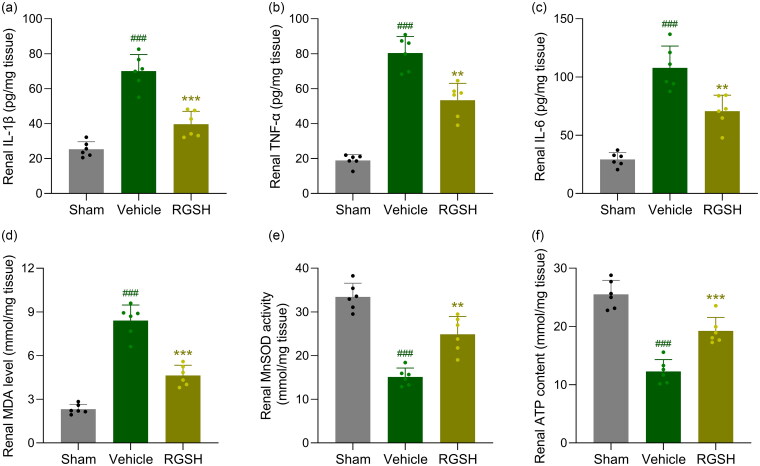
Reduced glutathione ameliorated renal inflammatory responses and mitochondrial-related oxidative stress in sepsis mice. Renal IL-1β (a), TNF-α (b) and IL-6 (c) at 24 h after CLP surgery were compared. MDA content (d), MnSOD activity (e) and ATP activity (f) in kidney tissues were determined at 24 h after CLP surgery. *n* = 6 for each group. ^###^*p* < 0.001 compared to sham and **p < 0.01, ***p < 0.001 compared to vehicle.

We further evaluated the effects of reduced glutathione on inflammation and oxidative stress in CLP mice by measuring the levels of pro-inflammatory cytokines IL-1β, IL-6, and TNF-α in kidney tissues. CLP mice exhibited significantly elevated levels of IL-1β (Figure 4a), TNF-α (Figure 4b), and IL-6 (Figure 4c) in kidney tissues. In contrast, treatment with reduced glutathione significantly reduced the levels of these pro-inflammatory cytokines in the kidneys of CLP mice.

Additionally, we observed a significant increase in malondialdehyde (MDA) levels (Figure 4d), along with decreased superoxide dismutase (SOD) activity (Figure 4e) and ATP levels in the kidneys of CLP mice (Figure 4f). Interestingly, treatment with reduced glutathione restored the levels of MDA, SOD, and ATP in kidney tissues.

These results demonstrate that reduced glutathione effectively ameliorates both inflammation and oxidative stress in CLP mice.

### Reduced glutathione suppressed cuproptosis in CLP mice

**Figure 5.**
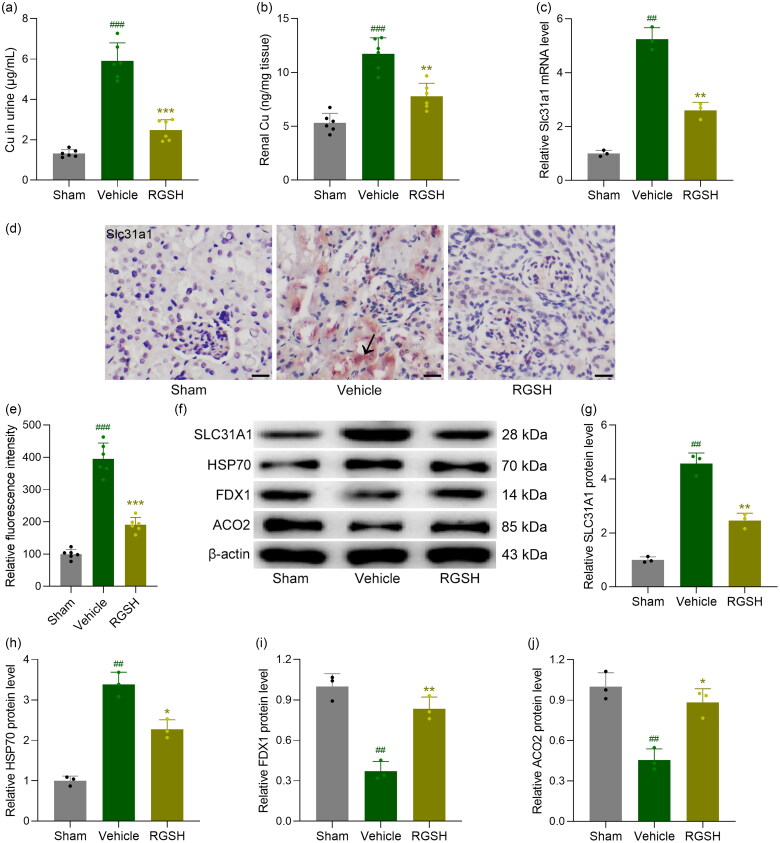
Reduced glutathione attenuated renal cuproptosis in sepsis mice. The Cu content in urine (a) and kidney tissues (b) were compared at 24 h after CLP surgery. The mRNA expressions of Slc31a1 in kidney tissues were detected by qRT-PCR (c). (d) Representative IHC staining of Slc31a1 with kidney tissues from each group (Arrow indicated the positive expression) and the relative fluorescence intensity was compared (e). The protein expressions of SLC31A1, HSP70, FDX1 and ACO2 in kidney tissues at 24 h after CLP surgery were measured by Western blotting (f). β-actin was used as a loading control and the expressions were normalized to Sham (g-j). Scale bar = 20 μm. *n* = 6 for each group. ^##^*p* < 0.01, ^###^*p* < 0.001 compared to sham and *p < 0.05, **p < 0.01, ***p < 0.001 compared to vehicle.

We next evaluated the effects of reduced glutathione on cuproptosis in CLP mice. CLP mice exhibited significantly elevated copper (Cu) levels in both urine (Figure 5a) and renal tissues (Figure 5b). In contrast, treatment with reduced glutathione dramatically decreased Cu levels in both urine and renal tissues of CLP mice.

Moreover, reduced glutathione treatment suppressed the mRNA expression of *Slc31a1*, a key factor in cuproptosis, in the kidney tissues of CLP mice (Figure 5c). To further elucidate the cellular localization of SLC31A1, we performed immunofluorescence staining on kidney sections (Figure S2). The results demonstrate that SLC31A1 is predominantly localized to the cell membrane of renal tubular epithelial cells. Correspondingly, immunohistochemistry (IHC) staining revealed increased expression of SLC31A1 in the kidney tissues of CLP mice (Figure 5d ande), whereas the expression of SLC31A1 was markedly reduced in CLP mice treated with reduced glutathione (Figure 5d ande). Western blot analysis further confirmed these findings at the protein level (Figure S3 and Figure 5f). Quantification of the blots showed that CLP significantly increased SLC31A1 protein expression, while reduced glutathione treatment attenuated this increase (Figure 5g). Additionally, we examined other markers of cuproptosis. HSP70, a marker of proteotoxic stress, was elevated in CLP mice and reduced by glutathione treatment (Figure 5h). Conversely, the iron-sulfur cluster proteins FDX1 and ACO2, which are typically depleted during cuproptosis, showed decreased expression in CLP mice, with levels partially restored by glutathione treatment (Figure 5i, j). These results collectively indicate that reduced glutathione effectively mitigates cuproptosis in the kidneys of CLP mice.

## Discussion

In the present study, we evaluated the effects of reduced glutathione on acute kidney injury and cuproptosis using a septic mouse model. Our findings demonstrated that reduced glutathione significantly improved survival rates, reduced bacterial burden, and suppressed both inflammation and oxidative stress in septic mice. Additionally, reduced glutathione ameliorated kidney function and injury. We further showed that reduced glutathione inhibited cuproptosis and the expression of SLC31A1, while promoting the expression of FDX1 and ACO2. Collectively, our study is the first to reveal that reduced glutathione ameliorates acute kidney injury in septic mice by inhibiting cuproptosis.

Glutathione (GSH) is the primary antioxidant in all tissues, existing mainly in two forms: reduced GSH and oxidized GSH (GSSG) [20]. The reduced form of GSH plays a central role in regulating various processes such as detoxification, protein folding, antiviral defense, and immune response [21]. Reduced GSH has been shown to suppress inflammation [22] and oxidative stress [23], two key factors involved in AKI [24,25]. In this study, we demonstrated that reduced GSH suppressed inflammation and oxidative stress in CLP septic mice, leading to an improved survival rate and rescued kidney function.

The protective effects of reduced GSH in sepsis and AKI have been previously described. Villa et al. used a mouse model of polymicrobial sepsis and found that GSH depletion led to an increase in bacterial colonies and worsened survival [26]. Conversely, treatment with the GSH precursor N-acetyl-L-cysteine reduced bacterial load and improved survival, highlighting the importance of GSH in the sepsis response. Additionally, He and colleagues reported that reduced GSH decreased BUN and serum MDA levels, while preventing glomerular injury and renal structural damage in a mouse model of AKI [16]. They further showed that reduced GSH inhibited iron accumulation and ferroptosis in AKI mice. Interestingly, Matsubara et al. established an AKI mouse model through systemic glutathione depletion, which strongly supported the essential role of GSH in protecting against kidney injury [27].

Cuproptosis is a novel form of programmed cell death triggered by the accumulation of Cu in mitochondria [28]. As an essential metal, Cu plays vital roles in numerous biological processes [29]. Under normal conditions, Cu levels are tightly regulated in the organism; however, excessive Cu triggers cuproptosis [30]. This process relies on the aggregation of lipoylated mitochondrial enzymes, which leads to mitochondrial stress and subsequent cell death. In our study, we observed significantly increased mitochondrial oxidative stress, along with markedly elevated Cu levels in both the kidneys and urine of CLP mice, suggesting the presence of cuproptosis in these mice. SLC31A1 is a critical modulator of Cu homeostasis and a key transporter for Cu uptake [31]. Elevated expression of SLC31A1 has been associated with AKI [11]. Consistent with this, we found significantly upregulated expression of SLC31A1 in the kidneys of CLP mice. In contrast, reduced GSH treatment suppressed SLC31A1 expression in CLP mice, which could account for the decreased Cu levels observed.

Cuproptosis is associated with the lipoylation of proteins in the tricarboxylic acid (TCA) cycle, leading to the accumulation of lipoylated proteins, the loss of iron-sulfur cluster proteins, and the expression of heat shock protein 70 (HSP70) [28]. Similarly, we observed increased expression of HSP70 in the kidneys of CLP mice, while reduced GSH treatment significantly reduced HSP70 levels.

Ferredoxin 1 (FDX1) is an upstream regulator of protein lipoylation and plays a crucial role in cuproptosis, as well as in the synthesis of iron-sulfur proteins [28,32]. Reduced FDX1 levels have been linked to various diseases, including cancers [33]. In our study, we identified a downregulation of FDX1 in CLP mice, while treatment with reduced GSH upregulated FDX1 expression. Furthermore, reduced GSH treatment rescued the expression of ACO2, an important enzyme in the TCA cycle, which is critical for maintaining iron homeostasis and oxidative stress defense [34]. Taken together, our findings demonstrate that reduced GSH effectively inhibits cuproptosis in CLP-induced AKI, likely through the modulation of key molecules involved in copper homeostasis and mitochondrial function.

Despite the valuable findings in this study, there are still several limitations. Although we demonstrated that reduced GSH suppressed cuproptosis and the expression of SLC31A1, the precise molecular mechanisms underlying these effects remain unclear. A study by Qiu et al. suggested that ELF3 transcriptionally activates SLC31A1 [11]. Future studies should investigate whether GSH suppresses SLC31A1 expression by modulating ELF3 and explore the effects of reduced GSH on cuproptosis-related diseases. Further elucidation of how GSH regulates SLC31A1, FDX1, and ACO2, and its effects on mitochondrial function in various contexts, will provide valuable insights into the intricate relationship between GSH and copper homeostasis. Moreover, since cuproptosis plays a critical role in the development of various diseases, it would be valuable to further explore the effects of reduced GSH on these diseases in future studies [35].

Our findings align with recent studies that have highlighted the importance of cuproptosis in various pathological conditions. For instance, Qiu et al. demonstrated that SLC31A1 plays a crucial role in cisplatin-induced AKI by triggering cuproptosis through mitochondrial dysfunction [11]. Similarly, in diabetic cardiomyopathy, AGEs were found to promote cuproptosis by upregulating SLC31A1 through ATF3/SPI1 signaling [36]. Furthermore, copper overload has been linked to cognitive dysfunction in mice, potentially through cuproptosis-mediated mechanisms [19]. These studies, along with our findings, underscore the widespread implications of cuproptosis in different organ systems and disease states. The ability of reduced GSH to modulate cuproptosis, as demonstrated in our study, suggests its potential therapeutic applications beyond AKI, possibly extending to other conditions where copper homeostasis is disrupted.

## Conclusions

In this study, we investigated the effects of reduced GSH on AKI in a CLP-induced septic mouse model. Our findings demonstrate that reduced GSH exerts protective effects by suppressing inflammation and oxidative stress, thereby rescuing kidney function in septic mice with AKI. Additionally, we identified that reduced GSH decreased Cu levels and prevented cuproptosis. This effect was likely mediated through the regulation of SLC31A1 expression, a copper transporter, which is correlated with cuproptosis. Overall, our study suggests that reduced GSH may represent a promising therapeutic approach for the treatment of AKI.

## Supplementary Material

Supplementary materials.docx

## Data Availability

Data are available upon reasonable request by contacting the corresponding author.
