# Supplementary material for: Reduced glutathione ameliorates sepsis-induced acute kidney injury in mouse model: involvement of cuproptosis
Source: Ren Fail. 2025 Jun 23;47(1):2518227. doi: 10.1080/0886022X.2025.2518227 (PMC12893487; doi:10.1080/0886022X.2025.2518227)
Supplement: Supplementary materials.docx [file IRNF_A_2518227_SM8842.docx]

**Supplementary materials**


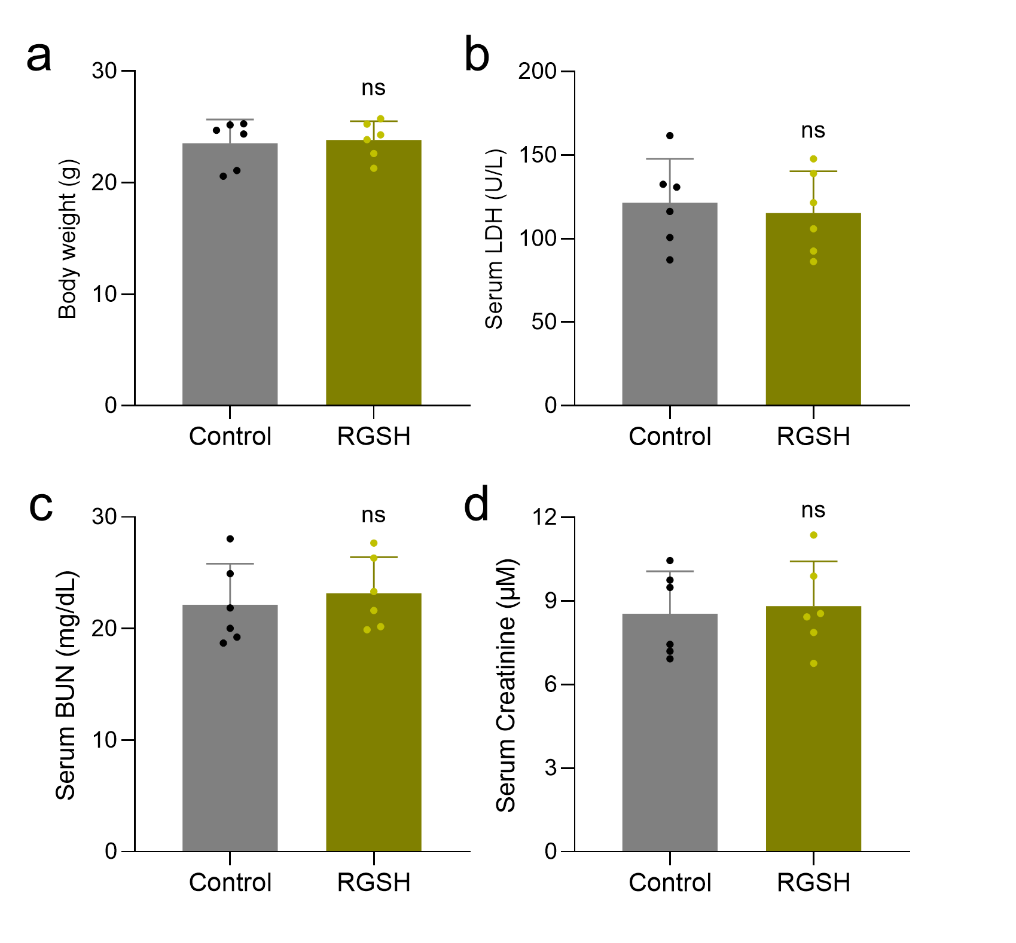


Figure S1. Reduced glutathione was safe to the healthy mice. Mice were intraperitoneally injected with 800 mg/kg RGSH daily for one week. The measurements of Body weight (a), serum LDH (b), BUN (c) and Creatinine (d). ns means no significance. Unpaired t test with Welch's correction.


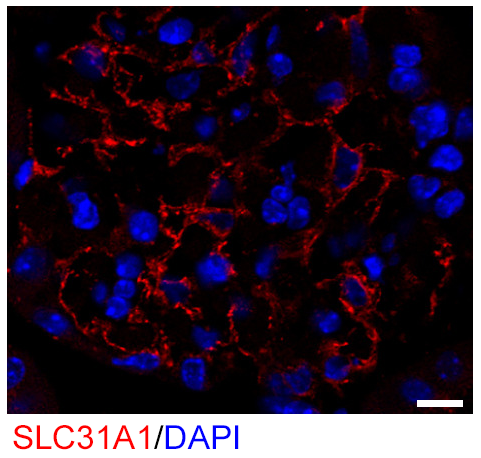


Figure S2. Immunofluorescent staining of SLC31A1expression in glomeruli of the mice.


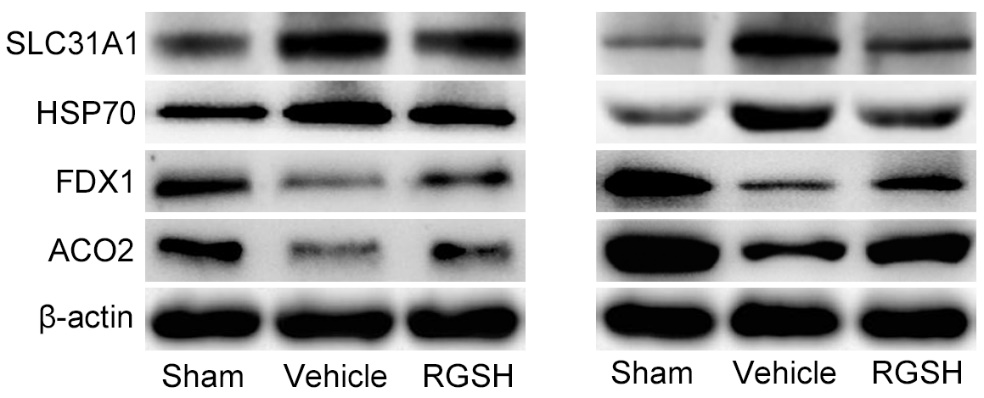


Figure S3. The other Western blots for Figure 5.
